# Surface clustering of metabotropic glutamate receptor 1 induced by long Homer proteins

**DOI:** 10.1186/1471-2202-7-1

**Published:** 2006-01-04

**Authors:** Paul J Kammermeier

**Affiliations:** 1 Department of Physiology and Pharmacology, Northeastern Ohio Universities College of Medicine, Rootstown, OH 44272, USA

## Abstract

**Background:**

Metabotropic glutamate receptors (mGluRs) regulate neuronal excitability and synaptic strength. The group I mGluRs, mGluR1 and 5, are widespread in the brain and localize to post-synaptic sites. The Homer protein family regulates group I mGluR function and distribution. Constitutively expressed 'long' Homer proteins (Homer 1b, 1c, 2 and 3) induce dendritic localization of group I mGluRs and receptor clustering, either internally or on the plasma membrane. Short Homer proteins (Homer 1a, Ania-3) exhibit regulated expression and act as dominant negatives, producing effects on mGluR distribution and function that oppose those of the long Homer proteins.

There remains some controversy over whether long Homer proteins induce receptor internalization by inducing retention in the endoplasmic reticulum, or induce mGluR clustering on the plasma membrane. Further, an exhaustive study of the effects of each long Homer isoform on mGluR distribution has not been published.

**Results:**

The distribution of a GFP-tagged group I mGluR, mGluR1-GFP, was examined in the absence of Homer proteins and in the presence of several Homer isoforms expressed in sympathetic neurons from the rat superior cervical ganglion (SCG) using total internal reflection fluorescence (TIRF-M) and confocal microscopy. Quantitative analysis of mGluR1-GFP fluorescence using TIRF-M revealed that expression of each long Homer isoform tested (Homer 1b, 1c, 2b and 3) induced a significant degree of surface clustering. Using confocal imaging, Homer-induced mGluR clusters were observed intra-cellularly as well as on the plasma membrane. Further, in approximately 40% of neurons co-expressing mGluR1-GFP and Homer 1b, intracellular inclusions were observed, but plasma membrane clusters were also documented in some Homer 1b coexpressing cells.

**Conclusion:**

All long Homer proteins examined (Homer 1b, 1c, 2b and 3) induced a significant degree of mGluR1-GFP clustering on the plasma membrane compared to cells expressing mGluR1-GFP alone. Clusters induced by long Homers appeared on the plasma membrane and intracellularly, suggesting that clusters form prior to plasma membrane insertion and/or persist after internalization. Finally, while Homer 1b induced surface clustering of mGluR1 in some cells, under some conditions intracellular retention may occur.

## Background

Group I metabotropic glutamate receptors (mGluR1 and 5) are phospholipase C linked G protein coupled receptors widely expressed in the mammalian nervous system [1]. Both mGluR1 and mGluR5 are often expressed near the post-synaptic density where they regulate synaptic strength by mediating several forms of synaptic plasticity [2-7] and directly modulating synaptic currents carried by NMDA [8,9] and non-NMDA ionotropic glutamate receptors [10,11].

The recently discovered Homer protein family [12] regulates both the distribution and function of group I mGluRs [13-15]. Constitutively expressed 'long' Homer proteins (Homer 1b, 1c, 2 and 3) possess a carboxy-terminal tail including a coiled-coil and two leucine zipper motifs [16-18]. This C-tail enables self-multimerization of the long Homer proteins, which act as scaffolds for its binding partners including group I mGluRs [12], IP_3 _receptors and ryanodine receptors [17], TRPC1 [19], the post-synaptic protein Shank [20] and others [21]. By aggregating these signaling proteins into clusters, Homer proteins appear to play a role in organizing efficient signaling domains [14].

Coupling of these proteins to effectors may be regulated by expression of the 'short' Homer proteins, Homer 1a and Ania 3 [12,22]. These isoforms lack the multimerizing C-tail, but bind proteins such as mGluRs similarly to the long Homers. Further, short Homer protein levels are regulated via immediate early expression [12], exhibiting elevated expression following periods of stress such as neuronal activity, seizures, tissue damage [23], and activation of certain signaling cascades [24,25]. Thus, a model has emerged to describe the regulation of mGluR signaling by Homer proteins in which long Homer proteins, constitutively expressed, organize group I mGluRs near the post-synaptic density or into somatic clusters that promote coupling to specific effectors such as IP_3 _receptors and ionotropic receptors. Following up-regulation of short Homers, the clusters are dispersed, disrupting coupling to effectors within these domains and promoting coupling to other effectors.

Many recent studies have specifically addressed the effects of Homer proteins on group I mGluR distribution. Induction of surface clusters of mGluRs has been reported with long Homer proteins [14,20,26,27]. Decreased surface expression of mGluRs has also been reported in clonal cell lines [28-30] and neurons [31], in some cases attributed to endoplasmic reticulum (ER) retention [30]. Other studies have reported mGluR clusters associated with long Homer protein expression without distinguishing surface or intracellular localization [16,32]. In intact neurons, long Homer proteins seem to promote dendritic and/or post-synaptic localization of mGluRs [26], while coexpression of Homer 1a is associated with a more general distribution. Taken together, these studies suggest an analogous relationship between somatic mGluR/Homer clusters and assembly around the post-synaptic density.

In the current study, the effect of several isoforms of long Homer protein as well as Homer 1a on the distribution of a GFP-tagged mGluR1 has been examined to assess whether each Homer protein promotes similar mGluR cluster formation in the absence of immuno-labeling, which may induce artifactual cluster formation. In addition, the sub-cellular localization of these clusters was assessed using total internal reflection fluorescence (TIRF-M) and confocal microscopy.

## Results and Discussion

### Plasma membrane clustering of mGluR1-GFP

**Figure 1:**
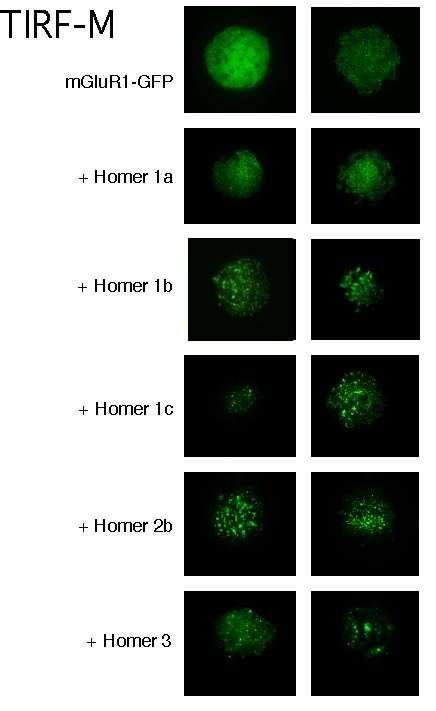
TIRF-M images of mGluR1-GFP fluorescence in SCG neurons expressing the receptor alone, "mGluR1-GFP", or with co-expression of the indicated Homer protein. Two representative neurons from each group are shown. The total number of neurons examined for each group were: mGluR1-GFP (19 cells), + Homer 1a (5), + Homer 1b (6), + Homer 1c (9), + Homer 2b (8), + Homer 3 (10).

To determine the effect of co-expression of each Homer protein isoform on the plasma membrane distribution of a group I mGluR, mGluR1-GFP was expressed alone or in combination with each of several Homer proteins in isolated sympathetic neurons from the rat SCG. TIRF-M images were taken of cells under each expression condition (Figure 1) to examine only plasma membrane-associated mGluR1-GFP. Neurons expressing either mGluR1-GFP alone or mGluR1-GFP plus Homer 1a exhibited a relatively diffuse distribution while cells expressing the long Homer isoforms Homer 1b, 1c, 2b and 3 exhibited various degrees of clustering (Figure 1).

Homer 2b induced the most pronounced surface clustering, though cluster formation was observed with each long Homer isoform. Cells co-expressing Homer 2b reliably showed large mGluR1-GFP clusters on the plasma membrane. Nearly all of the fluorescence in these cells appeared to be associated with a cluster. Further, similar large clusters were not observed in control or Homer 1a expressing neurons.

Although qualitatively, mGluR1-GFP distribution on the plasma membrane appeared more clustered in long Homer-expressing cells, the distribution and intensity of fluorescence in control and Homer 1a co-expressing cells was not absolutely uniform. In addition, not every cell expressing a long Homer protein showed obviously strong cluster formation. Therefore, an unbiased method was needed to quantify 'clustering' such that each group could be compared to determine if each long Homer protein altered mGluR distribution similarly.

Close inspection of the control TIRF-M images in Figure 1 (*top row*) shows that the distribution of receptor under this condition is not truly uniform, but organized in very small 'aggregations' that seem to be relatively diffusely distributed. These aggregations clearly differ from the very large, discreet clusters seen when long Homer proteins are co-expressed with mGluR1-GFP. It would also be necessary to distinguish true clusters from discreet regions of fluorescence resulting from apparently non-uniform contact of the plasma membrane with the cover slip (such as that in Figure 1, 'Homer 1a'; *second row*, *right*), resulting in patchy areas of fluorescence.

**Figure 2:**
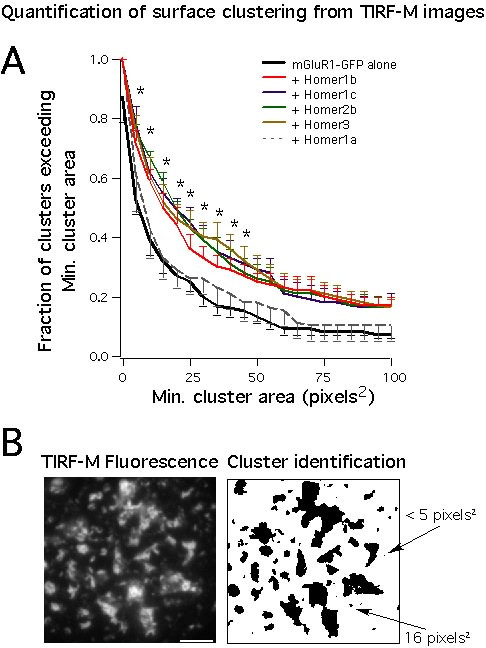
A, Cumulative histograms showing average (error bars indicate SEM) fraction of clusters in cells as a function of cluster size. The data analyzed were from the neurons described in Figure 1. [Asterisks indicate a statistically significant change from control for 1 or more long Homer protein groups.] B, An illustration of the particle analysis for a Homer 2b co-expressing cell. *Left *shows the image of the region of interest for this cell. *Right *shows the areas exceeding 1.5× the average fluorescence of the region of interest. 'Clusters' were defined as contiguous regions above this threshold. Two examples of clusters of various sizes are shown. Differences were seen in cluster size ranging from 10 to 50 pixels^2^. Asterisks indicate bins at which all long Homer groups differed from control and + Homer 1a cells. Scale bar is 5 μm.

Thus, cumulative histograms (Figure 2A) showing fraction of total clusters vs. cluster size were generated to quantitatively distinguish true Homer-induced clustering from mundane non-uniformities in the spatial distribution of mGluR1-GFP. For this analysis, clusters were defined as contiguous regions of fluorescence exceeding 1.5 times the average fluorescence of a large area within the cell (a rectangular region as large as possible excluding background). In particular, clusters ranging from 5 to 50 pixels^2 ^in area (~0.075 to 0.75 μm^2^; circular clusters ranging from about 0.3 to 1 μm in diameter) were examined (Figure 2A). Figure 2B illustrates a sample TIRF-M image from an mGluR1-GFP + Homer 2b expressing cell (*left*), and the regions exceeding 1.5× the average fluorescence of the illustrated selection are also illustrated (*right*). The figure indicates cluster of less than 5 pixels^2 ^(< 0.075 μm^2^; excluded from analysis in Figure 2A), and a 16 pixel^2 ^cluster (~0.24 μm^2^). This figure illustrates that this method is reasonably good at detecting mGluR1-GFP clusters. While several closely apposed clusters may be misidentified as a single large cluster, these are not counted within the critical range examined in Figure 2A. Therefore, the quantitative method used here includes some bias toward fewer clusters counted in highly clustered cells. Importantly, this method circumvents the two major problems encountered with cluster quantification, namely 1) misidentification of many very small, less intense aggregations as clusters and 2) misidentification of a large portion of a cell with diffuse receptor distribution as a very large single cluster. Thus, this method appears superior to simple cluster counting or measuring cumulative cluster size (the product of cluster number and average cluster area).

Cumulative histograms were thus generated from TIRF-M images obtained from SCG neurons in each expression group (indicated in the legend; Figure 2A), using a 5 pixel^2 ^bin size (± SEM). While the Homer 1a group was indistinguishable from control, each of the long Homer protein groups showed statistically greater clustering at one or more cluster sizes. Notably, a three-fold greater concentration of Homer 1b cDNA injected was required to produce statistically significant effects, compared to that used for the other Homer proteins. This may indicate that the construct is a poor expressor or less efficient at promoting clustering. Due to the nature of these experiments (injection and observation of a small number of cells), it was impractical to distinguish between these possibilities. These data indicate that co-expression most of the long Homer proteins tested with mGluR1-GFP induces receptor clustering on the plasma membrane to a greater degree than in cells expressing receptor alone.

### Subcellular distribution of mGluR1-GFP

**Figure 3:**
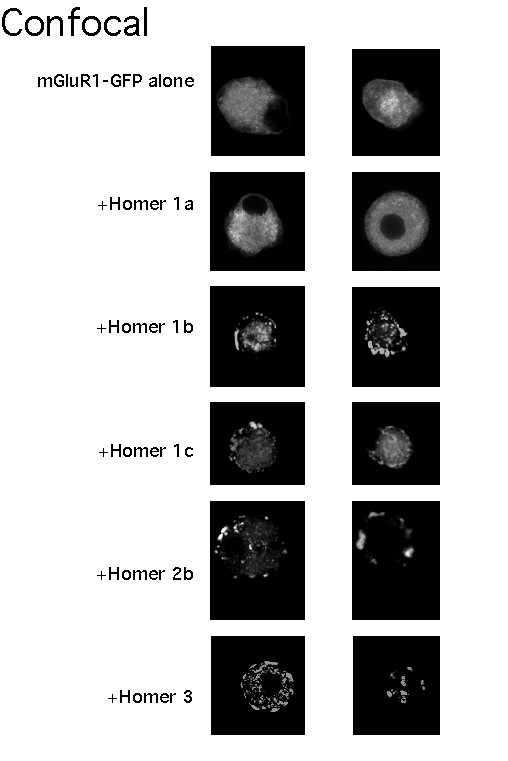
Confocal images of mGluR1-GFP fluorescence in SCG neurons expressing the receptor alone, "mGluR1-GFP", or with co-expression of the indicated Homer protein. Two representative neurons from each group are shown. The total number of neurons examined for each group were: mGluR1-GFP (26 cells), + Homer 1a (9), + Homer 1b (12), + Homer 1c (12), + Homer 2b (34), + Homer 3 (7).

To examine the subcellular distribution of mGluR1-GFP and mGluR1-GFP clusters in the presence of long Homer proteins, neurons expressing these proteins were examined using confocal microscopy. Figure 3 shows representative sample confocal images of SCG neurons expressing mGluR1-GFP alone or in conjunction with each of the Homer proteins indicated. The control (*top row*) and '+ Homer 1a' (*second row*) images illustrate that while receptor distribution is not absolutely uniform, the receptors are distributed fairly diffusely. Further, in control and with every Homer protein examined, the large majority of the receptors present in these neurons resided intracellularly. That is, only a small fraction of the total mGluR1-GFP in each cell was present on the plasma membrane, available to be activated by agonist.

As with the TIRF-M images in Figure 1, the confocal images clearly show that co-expression of the long Homer proteins correlates with the appearance of large mGluR1-GFP clusters. In most cases, clustering of mGluR1-GFP appeared equally strong when the receptors were localized intracellularly, akin to those on the surface (see Figures 1 and 3). This may be an indication that Homer-induced mGluR clusters assemble relatively early, perhaps prior to insertion into the plasma membrane, and may also remain assembled as large clusters following internalization. However, confirmation of these conclusions will require further study.

**Figure 4:**
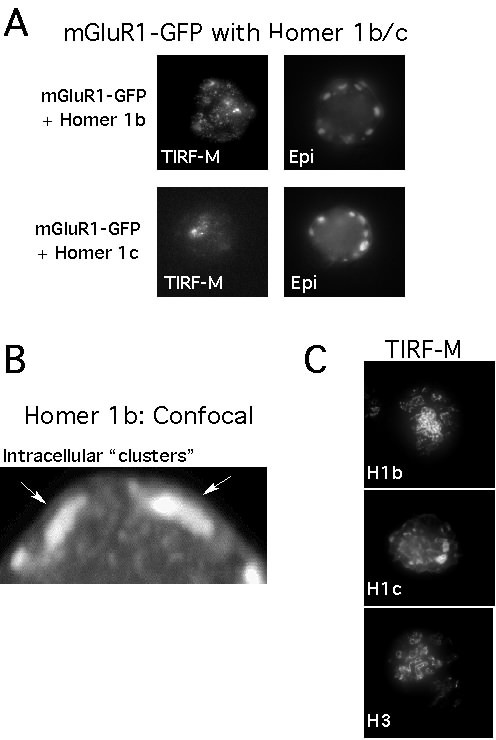
Differential cluster formation in SCG neurons expressing mGluR1-GFP with Homer 1b. A, Examples of Homer 1b and 1c co-expressing cells with surface clustering (TIRF; *left*), and apparent intracellular aggregations of mGluR1-GFP (Epifluorescence; *right*). B, Expanded views of a confocal image showing intracellular aggregations of receptor clusters taken from a Homer 1b co-expressing cell. C, Anomalous mGluR1-GFP distribution induced by Homer proteins indicated in a small number of TIRF-M images.

A reduction of surface expression of group I mGluRs in conjunction with Homer 1b expression has been reported in HEK293 cells [29,30,33] and in neurons [31]. Quantifying plasma membrane receptor as a fraction of total receptor using confocal imaging is difficult in this case since, as noted above, a large majority of the total receptor resides intracellularly regardless of the Homer protein present. However, in 5 of the12 Homer 1b coexpressing neurons for which confocal images were obtained (~40%), large inclusions were evident, apparently just below the plasma membrane. These inclusions appeared qualitatively similar to those observed in previous studies in neuronal and non-neuronal cells [16,31] in which Homer 1b induced retention of group I mGluRs. Figure 4A, *upper left*, shows an example of a Homer 1b-expressing neuron with clustering similar to that observed with other long Homer proteins. Figure 4A, *upper right*, illustrates the large, intracellular inclusions in the same neuron taken under epifluorescence. Similar inclusions were observed in one Homer 1c-expressing neuron (Figure 4A, *lower*). TIRF-M and epifluorescence images are shown from this cell, illustrating a small number of surface clusters and large intracellular inclusions.

Figure 4B, shows an expanded view of a confocal image of a Homer 1b coexpressing cell illustrating the large, bright structures beneath the plasma membrane. These data suggest that Homer 1b may promote a decrease in mGluR1 surface expression, perhaps via an ER retention mechanism similar to that previously reported [30]. This kind of receptor distribution was not observed in confocal images of cells expressing other isoforms of Homer protein, including Homer 1a (9 cells), Homer 2b (34 cells), or Homer 3 (7 cells), nor were such structures observed in cells expressing mGluR1-GFP alone (26 cells). Similar structures were not seen in any of 12 confocal images taken from Homer 1c-coexpressing neurons, but as noted above, similar inclusions were observed in one Homer 1c-expressing cell used for TIRF-M imaging (Figure 4A). Interestingly, the TIRF-M images of the cells in Figure 4A revealed that clustering was observed on the plasma membrane in these cells in which intracellular inclusions were also seen. In the Homer 1c example, despite verification of total reflection, some out-of-focus light is apparent. This presumably occurred because the intensity of the intracellular clusters of mGluR1-GFP in this cell far exceeded that on the cell surface. Therefore, even though the evanescent wave illuminating this neuron decayed exponentially with distance from the cover slip, some fluorescence beneath the membrane was detectable. These data show that in cell in which mGluR1 surface expression is reduced by Homer coexpression, the mGluRs remaining on the surface are indeed organized in clusters.

### Anomalous effects

While most neurons examined exhibited either diffuse receptor distribution or some degree of clustering qualitatively similar to the examples shown above, TIRF-M imaging revealed that a small number of cells (1–2 from each group) from Homer 1b, 1c and 3 cells exhibited an anomalous distribution pattern (Figure 4C). While the distribution of mGluR1-GFP in these cells is clearly non-uniform, close inspection reveals that the receptor is not organized into clusters that resemble those 'normally' observed with TIRF-M (Figure 1). It is unclear whether these neurons represent a separate manifestation of the effects of Homer protein expression, or simply an early (or late) stage of the clustering (plasma membrane or intracellular) normally observed. Unfortunately, the rare occurrence of this anomalous effect has thus far precluded careful examination.

The data presented here demonstrate that the long Homer proteins Homer 1b, 1c, 2b and 3 organize mGluR1 into plasma membrane clusters to a significant degree. Further, neurons expressing Homer 1b and to a lesser extent, Homer 1c, appeared to induce some internalization or retention of mGluR1, consistent with previous reports. This study shows for the first time a side-by-side comparison of plasma membrane mGluR1 clustering induced by several Homer protein subtypes, and demonstrate that each long Homer protein tested appears to produce mGluR1 surface clustering.

## Methods

A detailed description of the cell isolation and cDNA injection protocol is published elsewhere [34]. Animal protocols were approved by our Institutional Animal Care and Use committee (IACUC). Briefly, both SCGs were removed from adult Wistar rats (175–225 g) following decapitation, and incubated in Earle's balanced salt solution (Life Technologies Inc., Rockville, MD) containing 0.55 mg ml^-1 ^trypsin (Worthington Biochemicals, Freehold, NJ) and 0.7 mg ml^-1 ^collagenase D (Boehringer Mannheim Biochemicals, Indianapolis, IN) for 1 hour at 35°C. Cells were then centrifuged (50 × G), transferred to minimum essential medium (Fisher Scientific, Pittsburgh, PA), plated on poly-L-lysine coated 35 mm glass-bottom tissue culture dishes (World Precision Instruments, Sarasota, FL; MatTek, Ashland, MA) and incubated (95% air and 5% CO_2_; 100% humidity) at 37°C prior to DNA injection. After injection, cells were incubated overnight at 37°C and imaging experiments were performed the following day.

Transfection of SCG neurons was done by intra-nuclear injection using an Eppendorf FemtoJet microinjector and Injectman NI2 micromanipulator (Madison, WI) 4–6 hours following cell isolation. Plasmids were stored at -20°C as a 1 μg μl^-1 ^stock solution in TE buffer (10 mM TRIS, 1 mM EDTA, pH 8). The mGluR1-GFP plasmid (obtained from Stephen R. Ikeda, NIH; rat mGluR1 sequence), a fusion protein with GFP on the C-terminal tail of the full length rat mGluR1, was injected at 50–70 ng μl^-1 ^(pEGFP_N1_; Clontech, Mountain View, CA). Homer cDNAs (pRK5; obtained from Paul F. Worley, Johns Hopkins University) were injected at 100 ng μl^-1^, with the exception of Homer 1b, which was injected at 300 ng/μl, as noted in the text.

Inserts were sequenced using an automated DNA sequencer (CQ2000, 8 channel capillary DNA sequencer, Beckman Coulter, Fullerton, CA). PCR products were amplified and purified with Qiagen (Valencia, CA) anion exchange columns. Plasmids were propagated in Top10 bacteria (InVitrogen, Carlsbad, CA).

TIRF-M images were acquired on an Olympus IX-71 microscope equipped with a 60×, 1.45 N.A., oil-immersion objective and a single-line (488 nm), Argon ion laser. TIRF mode was verified by two methods: 1) changing the focal plane to insure that all visible fluorescence was contained within a single focal plane, and 2) by examining the laser light using the microscope's centering telescope. Images were acquired with a Spot-RT, cooled CCD camera (Diagnostic Instruments, Sterling Heights, MI), generously loaned by Stephen R. Ikeda (NIAAA, NIH, Rockville, MD). Confocal images were obtained using an Olympus IX-70 Fluoview scanning confocal microscope equipped with a single-line, 488 nm Argon ion laser and a dual-line Krypton ion laser. All image analysis was performed using Igor Pro software (Wavemetrics Inc., Lake Oswego, OR).

Cluster analysis was done using TIRF-M images by generating cumulative histograms of cluster number *vs*. size. A rectangular region was selected within the cell fluorescence area. Regions of fluorescence not exceeding 1.5× the average of the selected area were discarded, and particle analysis was performed on the remaining areas (see Figure 2B). Each contiguous particle larger than the minimum area of pixels, *m*, was counted, then *m *was increased by 5 (up to 1000), and particles recounted at each iteration to generate the cumulative histogram. All images were obtained using the same magnification, gain, etc. such that a pixel corresponded to a square region, approximately 123 nm on a side (about 8.1 linear pixels per μm). Thus, the analysis eliminates consideration of bright regions < 0.075 μm^2 ^(circular regions < ~0.3 μm in diameter). Statistical analysis (non-parametric ANOVA with Dunn post-hoc test performed for data at each bin; p ≤ 0.05 was used to define significance) was performed with InStat software (Graphpad Software, San Diego, CA).
